# Grain Size-Dependent Defect and Domain Evolution in Lead Titanate-Based Relaxor Ferroelectrics

**DOI:** 10.1021/acsami.6c00336

**Published:** 2026-04-22

**Authors:** Hangfeng Zhang, Yichen Wang, Zilong Li, Soyoung Oh, Junjie Liu, Haixue Yan, Yang Hao, Lei Su

**Affiliations:** † School of Engineering and Material Science, 4617 Queen Mary University of London, Mile End Road, London E1 4NS, U.K.; ‡ State Key Laboratory of Powder Metallurgy, 12570 Central South University, Changsha 410083, China; § Department of Physics, 6396 University of Oxford, Oxford OX1 3PU, U.K.; ∥ School of Physical and Chemical Science, Queen Mary University of London, Mile End Road, London E1 4NS, U.K.; ⊥ School of Electronic Engineering and Computer Science, Queen Mary University of London, Mile End Road, London E1 4NS, U.K.

**Keywords:** domain engineering, grain size control, relaxor ferroelectrics, spark plasma sintering, piezoelectric

## Abstract

Ferroelectric materials are widely used in diverse applications, where their performance is strongly dominated by grain size. Here, dense Er-doped lead titanate-based relaxor ferroelectrics were synthesized via spark plasma sintering, enabling precise grain size control from 0.9 to 11.1 μm. Fine-grained ceramics exhibit high defect concentrations and internal stress, stabilizing the tetragonal phase and resulting in weak, disordered polarization with low domain wall density and constrained mobility. At intermediate grain sizes, dense nanodomain networks with narrow walls (∼150 nm) form, allowing sharp and reversible polarization switching. Coarse-grained ceramics develop hierarchical, web-like domains with thicker walls (∼400 nm), reducing the pinning effect and enhancing wall mobility. Both saturation and remanent polarizations increase with grain size up to 5.4 μm before plateauing, while the piezoelectric coefficient rises by 200%, reaching 723 pC N^–1^. These results demonstrate grain-size engineering as an effective route to optimize domain wall structure and relaxor ferroelectric performance.

## Graphical abstract

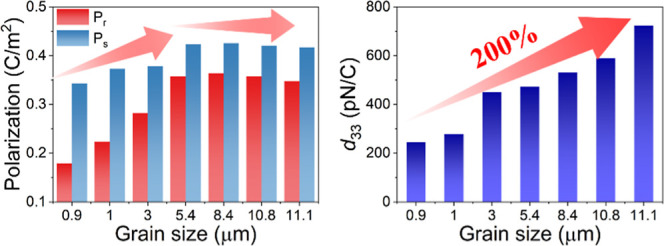

## 1 Introduction

Ferroelectric materials are characterized by an inherent switchable spontaneous polarization under an external electric field. Their paraelectric phases are widely used in high-power capacitors and wireless communication devices (e.g., Ba_1–*x*_Sr_*x*_TiO_3_ used as a paraelectric tunable dielectric in the RF/microwave range).^1–4^ In ferroelectric ceramics, the dielectric response arises from both intrinsic and extrinsic contributions. Intrinsic contributions originate from the lattice-level structure associated with chemical compositions.^5^ Extrinsic contributions are from point defects, twin boundaries, including domain configuration, phase boundary interactions, internal stress, and grain boundaries, which significantly affect dielectric and piezoelectric behavior.^6–10^ The effect of grain size on crystal structure and dielectric and piezoelectric properties has been extensively studied in several typical ferroelectric ceramics.^11,12^

In classical ferroelectrics such as BaTiO_3_, Arlt et al. demonstrated that, within the micrometer grain-size range, dielectric and piezoelectric properties are strongly influenced by domain configuration and domain wall contributions.^13–15^ The domain width was shown to be proportional to the square root of the grain size, and dielectric permittivity reaches a maximum at approximately 0.8–1 μm. When the grain size decreases below ∼700 nm, reduced tetragonality and suppressed domain wall contributions lead to a significant reduction in dielectric response.^14^ This behavior has been interpreted as a critical grain-size effect associated with the transition toward single-domain or pseudosingle-domain structures.^6^ Later in situ high-energy X-ray diffraction studies provided direct experimental evidence that internal residual stress does not vary significantly with grain size. Instead, enhanced 90° domain wall displacement was the main contributor for the improved dielectric and piezoelectric properties at intermediate grain sizes.^16^ In Pb­(Zr, Ti)­O_3_ (PZT) near the morphotropic phase boundary (MPB), a different grain-size dependence has been reported. Randall *et al.* showed that in the micrometer range (∼3–10 μm), domain width increases approximately with the square root of grain size, whereas in the submicron regime, the observed domain widths become smaller than predicted by this relationship, due to enhanced grain-boundary clamping and reduced domain variants, which strongly restrict domain-wall mobility. In contrast, sodium niobate ceramics with micrometer-sized grains and nanosized grains exhibit antiferroelectric and ferroelectric structures, respectively.^7,17^ In sodium niobate ceramics, smaller grains tend to form a ferroelectric structure that is energetically stable, whereas larger grains favor the formation of an antiferroelectric structure to minimize the free energy within the system. Unlike BaTiO_3_, where grain-size effect directly shifts phase transition point (*T*_c_), relaxors exhibit a broad dielectric maximum at *T*_m_ that does not correspond to a true thermodynamic phase transition. In relaxor systems such as Pb­(Mg_0.33_Nb_0.67_)_1–*x*_Ti_*x*_O_3_ (PMNT, *x* = 0.07), grain-size effects in micrometer scale range have been reported to only influence the magnitude and diffuseness of the dielectric maximum peaks (*T*_m_).^18^ Using a modified brick-wall model with a ∼2 nm intergranular glassy layer, it was shown that the observed grain-size dependence originates from extrinsic dielectric mixing effects rather than intrinsic changes in polarization dynamics. In relaxors, the true phase-related transition occurs at higher temperatures near the Burns temperature (*T*_B_), where polar nano regions (PNRs) first nucleate, or near the freezing temperature (*T*_f_), where dynamic polarization fluctuations slow down. Recent work on Bi_0.5_Na_0.5_TiO_3_-based relaxor ferroelectrics has demonstrated that grain size increase leads to a transition from ergodic relaxor behavior to more stable ferroelectric states by enlarging domain size and enhancing polar ordering.^19^ Thus, understanding and manipulating the grain-size effect is crucial for optimizing the performance of ferroelectric ceramics.

Lead titanate-based relaxor ferroelectric PMNT (*x* = 0.29–0.35), located near the MPB, is well-known for its high piezoelectric response, particularly in single crystals.^20^ In polycrystalline ceramic systems, the increasing piezoelectric performance is often achieved by lowering *T*_m_ or rhombohedral to tetragonal phase transition toward room temperature, which decreases the thermal stability of piezoelectric applications.^21^ By varying the titanium content, PMNT exhibits different domain structures, including low-angle nanodomain walls, which contribute to the high piezoelectric performance in relaxor ferroelectrics.^22^

The introduction of rare earth elements, such as samarium, europium, and neodymium, into PMNT has resulted in exceptionally high piezoelectric coefficients due to such chemical modifications allowing leveling of the thermodynamic energy states of various polar phases in the system.^2,23,24^ Our previous studies have shown that Er-doped PMNT, Er_0.025_Pb_0.9625_(Mg_0.33_Nb_0.67_)_0.7_Ti_0.3_O_3_ (ErPMNT), exhibits ultrahigh field-induced strain, demonstrating a field-induced phase transition with a mixed tetragonal and orthorhombic phase structure.^25^ However, conventional furnace-based preparation of PMNT-based ceramics can lead to issues such as lead volatilization, unwanted secondary phase formation, and low relative density during high-temperature sintering. In contrast, spark plasma sintering (SPS) offers an electric field-assisted rapid sintering process capable of simultaneous uniaxial pressure loading and high heating rate, enabling the densification of ceramic powders within minutes at lower temperatures.^26^ By careful control of heating rate, sintering temperature, and time, dense ceramics with various grain sizes can be prepared by SPS with minimum lead loss. Although Er-substitution may exhibit different site occupancies in BaTiO_3_ ceramics, B-site doping has previously been reported to induce an acceptor effect.^27^ However, such an acceptor effect was not observed in the present study. In this work, an Er dopant was designed to predominantly occupy the A-site, and all ceramics were prepared with identical compositions to ensure that the observed variations were primarily associated with grain-size effects.

Ferroelectricity is a long-range ordered phenomenon that is extremely sensitive to the grain size. Grain size reduction can significantly influence polarization stability, domain-wall dynamics, and the overall performance of ferroelectric devices. Understanding these effects is crucial for both the miniaturization of ferroelectric components and insights into internal stress, domain, and phase interactions. Furthermore, the influence of grain size on dielectric properties, piezoelectric response, and domain wall behavior in rare-earth (e.g., Er)-doped lead titanate-based relaxor ferroelectrics has not been fully explored. In this work, ErPMNT ceramics with varying grain sizes were prepared by solid-state methods followed by SPS sintering. ErPMNT exhibited coexistence of tetragonal and orthorhombic structures, with a higher tetragonal phase fraction in smaller grain-sized ceramics. A comprehensive study was conducted to investigate the relationship between domain structure and macroscopic dielectric, ferroelectric, and piezoelectric properties, providing valuable insights for domain engineering through grain size manipulation to achieve optimum performance.

## 2 Results and Discussion

Scanning electron microscopy (SEM) images of the fracture surfaces of the studied ErPMNT ceramics show a dense morphology with an average grain size ranging from 0.9 to 11.1 μm (Figure S1). The average grain size gradually increased for ceramics sintered at 900 and 950 °C, followed by a rapid increase for ceramics sintered at higher temperatures up to 1150 °C. In addition, the ceramic sintered at 1150 °C for 5 min showed only a marginal increase in grain size compared to those sintered for 2 min. The relative densities of ceramics are all above 99%, with theoretical density determined from X-ray powder diffraction (XRD) refinement results. XRD patterns of ErPMNT sintered between 900 and 1150 °C for 2 min and 1150 °C for 5 min show almost identical diffraction peaks (Figure S2). In contrast, ceramics sintered at 1150 °C for 10 min and 30 min and 1200 °C for 2 min, exhibit an additional secondary phase, identified as cubic pyrochlore Pb_3_Nb_4_O_13_, likely due to the excess lead loss (Figure S3).^28^ XRD analysis shows that ceramics sintered up to 1150 °C/5 min remain single phase within the XRD detection limit, while a minor pyrochlore phase appears only at higher temperatures or longer dwell times. Thus, 1150 °C/5 min represents an optimized SPS condition, balancing grain growth while minimizing secondary phase formation. A previous study on PMNT compositions in the MPB region, revealed the coexistence of orthorhombic (O phase) and tetragonal (T phase) phases at room temperature.^29–31^ Rietveld refinement was performed using fine powders, and no clear preferred orientation was observed in the refinement results. A two-phase model with a tetragonal structure in space group *P*4*mm* and an orthorhombic structure in space group *Amm*2 offers a better fit for XRD patterns compared to single-phase (orthorhombic or tetragonal) fittings (Figure 1). Close examination of the 2θ diffraction peaks at *ca.* 45°, small grain-sized ceramics reveal additional peaks (marked by arrows) attributed to the T phase reflections (002) and (200), which cannot be fitted by using single-phase model. These peaks become less pronounced as the grain size increases. The details of the fitted parameters and crystal structure are summarized in Table S1. Specifically, ceramics with grain sizes of 0.9 and 1.0 μm exhibit T phase fractions of approximately 40.5 and 38.5%, respectively. T phase fraction dramatically decreases to 15.6% for a 3.0 μm grain size ceramic and gradually decreases with larger grain size ceramics. The decrease in the tetragonal phase fraction with increasing grain size is attributed to the reduction in grain boundary constraints and associated internal stresses. Near the MPB, the free energy difference between tetragonal and orthorhombic phases is relatively small.^2^ In smaller grains, grain boundaries induce local stress fields that hinder the transformation to the orthorhombic phase upon cooling,^32^ thereby preserving a large amount of tetragonal phase in the system. By contrast, in larger grains, the residual stress is relieved, allowing the materials to relax into the more distorted orthorhombic phase.

**Figure 1:**
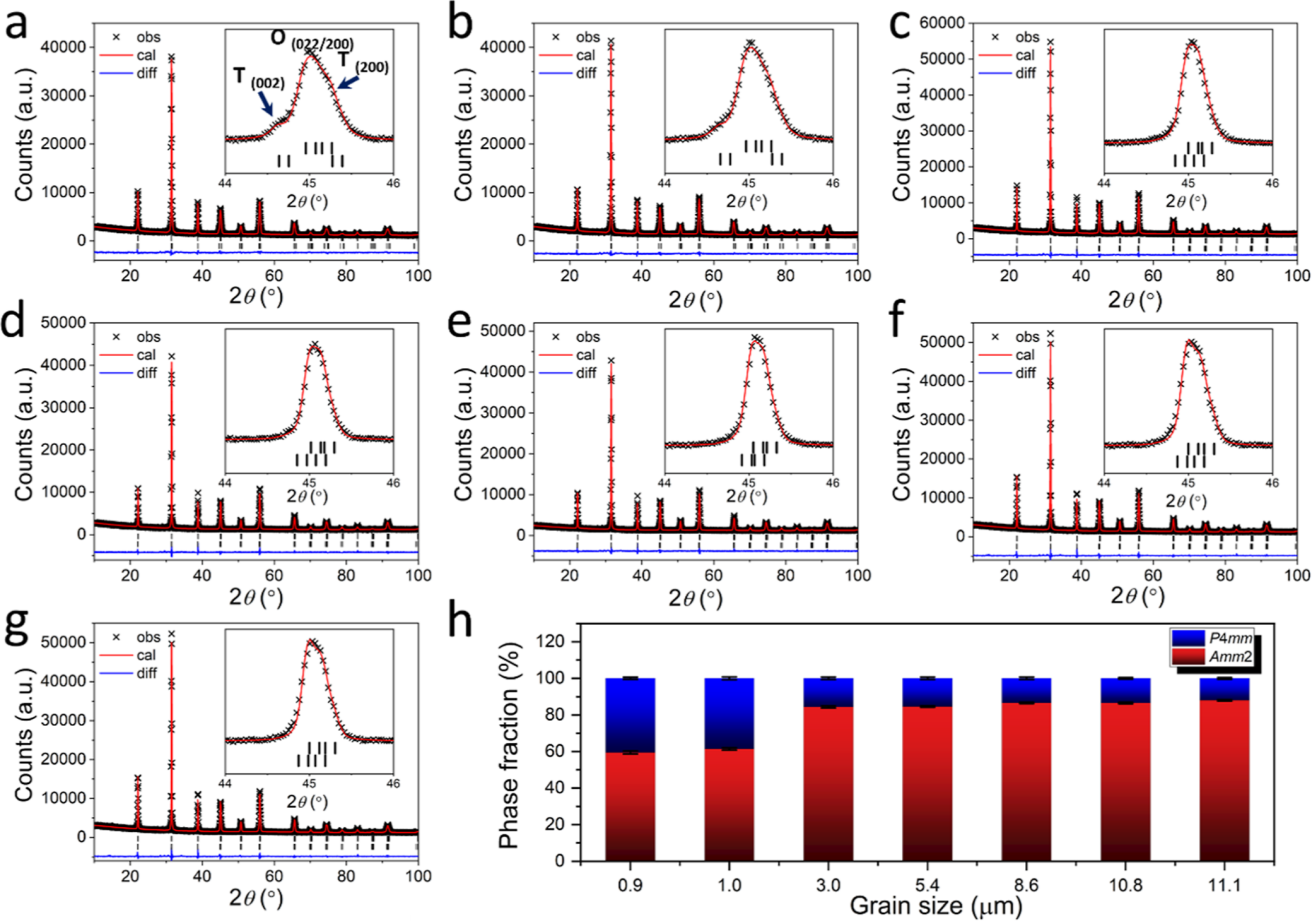
Fitted XRD profiles for ErPMNT ceramics with different grain sizes, (a) 0.9 μm, (b) 1.0 μm, (c) 3.0 μm, (d) 5.4 μm, (e) 8.6 μm, (f) 10.8 μm, and (g) 11.1 μm. (h) The phase fraction with error bar for studied composition with different grain sizes. Black bars mark the Bragg reflection positions of the tetragonal (*P*4*mm*, lower) and orthorhombic (*Amm*2, upper) phases.

The dielectric permittivity and loss tangent of all ceramics show little frequency dependence over the measured temperature range (Figure 2a–g). Moreover, there is no noticeable difference in dielectric permittivity at temperatures below 0 °C. Large grain-sized ceramic (>1 μm) exhibits a distinct dielectric permittivity peak *T*_m_ at *ca.* 150 °C. Another anomaly in dielectric permittivity occurs at *ca.* 120 °C, which is associated with the orthorhombic to tetragonal phase transition (Figure 2g). The maximum permittivity temperature difference (Δ*T* = *T*_m,500Hz_ – *T*_m,100kHz_) increases with grain size. It was noticed that larger grain-sized ceramics show lower *T*_m_ points but higher maximum dielectric permittivity (Figure 2h). The observed increase in *T*_m_ for smaller grains can be attributed to several factors: (i) the high grain boundary density acts as a dead layer, restricting domain motion; (ii) internal stresses that may enhance ferroelectric phase stability; and (iii) phase coexistence near MPB, which affects the stability of the tetragonal and orthorhombic phases. In BaTiO_3_, reducing grain size to the nanoscale weakens ferroelectric stability, resulting in a decrease in the Curie temperature and spontaneous polarization due to reduced tetragonality and suppression of long-range order.^33^ In contrast, relaxor ferroelectrics such as PMN and PMN–PT exhibit grain-size effect primarily in the magnitude and diffuseness of the dielectric maximum *T*_m_. Grain reduction suppresses low-frequency relaxations associated with PNR dynamics near grain boundaries, which lead to a broadening of *T*_m_.^34^ In contrast, small-grain ceramics (≤1 μm) show dielectric permittivity curves that remain flat over the 120–200 °C temperature range.

**Figure 2:**
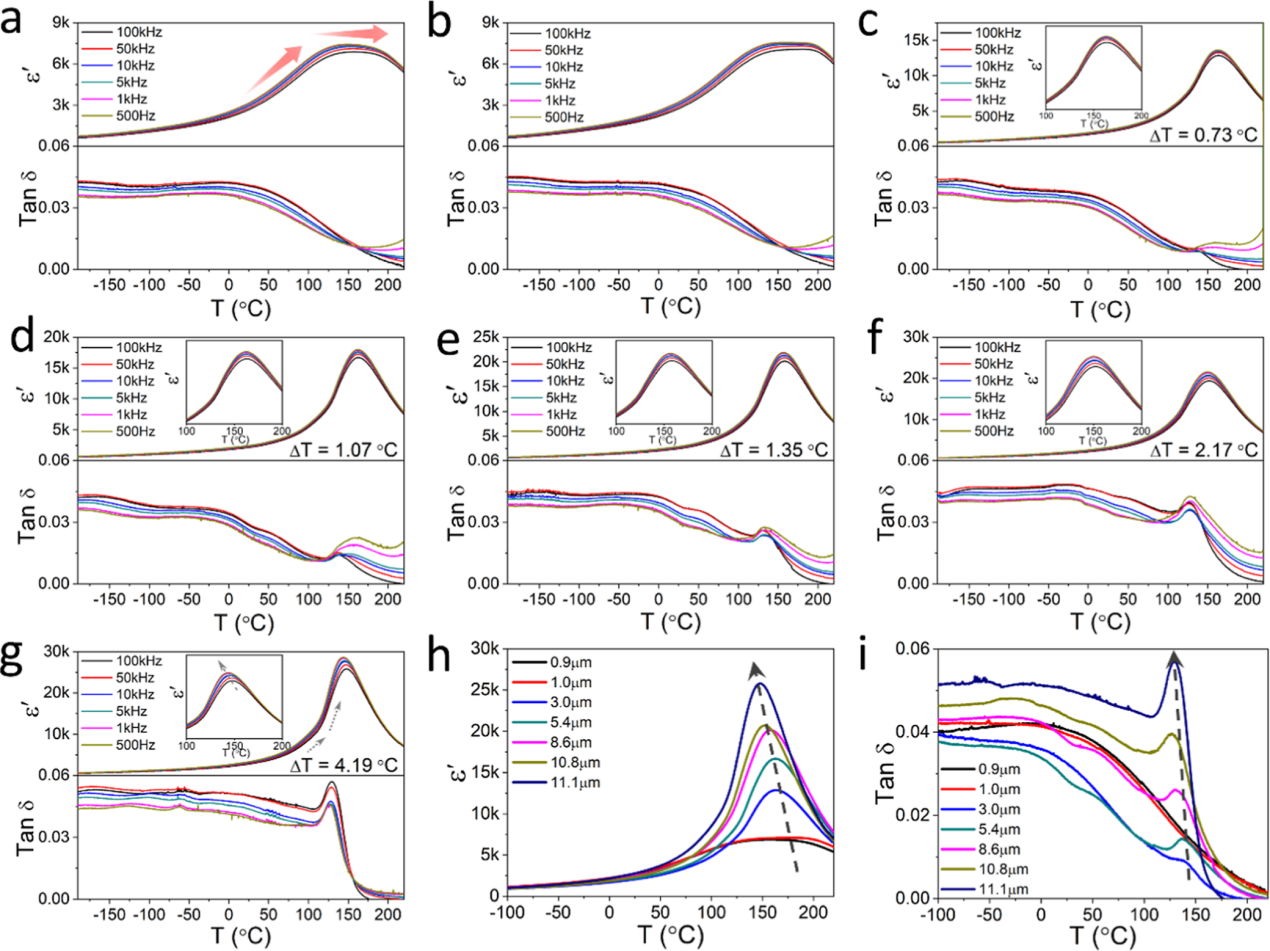
Temperature dependence of dielectric permittivity and loss measured at selected frequencies for ErPMNT ceramics with different grain sizes, (a) 0.9 μm, (b) 1.0 μm, (c) 3.0 μm, (d) 5.4 μm, (e) 8.6 μm, (f) 10.8 μm, and (g) 11.1 μm. Temperature dependence of (h) dielectric permittivity and (i) dielectric loss at 100 kHz for studied ErPMNT ceramics.

The flat permittivity curve is associated with two-phase transitions in the system, from the orthorhombic to the tetragonal and then to the cubic phase.^35^ The suppression of the dielectric peak and reduction in peak permittivity in smaller grain-sized ceramics suggest a diffuse phase transition from tetragonal to cubic, where internal stresses and grain boundary constraints disrupt long-range ferroelectric ordering, leading to the formation of PNRs. This leads to a more gradual phase transition, resulting in a broader and less-pronounced dielectric peak.^36^ The large tetragonal distortion (higher *c*/*a* ratio from XRD results) in smaller grains is likely due to internal stress, which stabilizes the tetragonal phase with enhanced lattice distortion, resulting in a higher *T*_m_. In contrast, large-grain-sized ceramics exhibit larger ferroelectric domains with long-range order, favoring the formation of the orthorhombic phase at room temperature. The reduction in internal stress in larger grains allows for a more abrupt phase transition, leading to a sharper dielectric peak and higher permittivity.

Regarding the dielectric loss spectra, small-grain ceramics exhibit a decrease in dielectric loss at temperatures above *ca.* 50 °C. In contrast, for large grain-sized ceramics, distinct dielectric loss peaks observed at *ca.* 140 °C become more pronounced with increased grain size. Dielectric loss also increases in large grain-sized ceramics, with the loss peak shifting to a lower temperature (Figure 2i). This phenomenon is associated with the reduced domain wall activity in the smaller grain-sized ceramics.^10^ Comparison of dielectric properties during heating and cooling reveals that small-grain sample exhibit minimal thermal hysteresis (Figure S4). With increasing grain size, the separation between heating and cooling spectra becomes more pronounced, which suggests increased ferroelectric distortion.

The current density-electric field (*J*–*E*) and polarization-electric field (*P*–*E*) measurement on ErPMNT ceramics reveals typical ferroelectric hysteresis loops with butterfly strain-electric field (*S*–*E*) loops (Figures 3a–f and S5). Notably, small grain-sized (≤1 μm) ceramics exhibit broad current peaks and higher coercive fields (*E*_c_, *ca.* 0.71 kV mm^–1^), indicative of constrained domain wall motion due to increased boundary pinning and internal stress. In contrast, large grain-sized ceramics show sharper current peaks and lower *E*_c_ (*ca.* 0.71 kV mm^–1^) compared to large grain-sized ceramics (*ca.* 0.53 kV mm^–1^) (Figure S5e), suggesting the higher domain wall pinning energy at smaller grain-sized ceramics. Both saturation (P_s_) and remanent (P_r_) polarization of the ceramics increased with grain size and then level off beyond 5.4 μm, suggesting enhanced domain alignment and increased switchable domain volume, associated with the reduced pinning effect and phase coexistence at MPB.^10^ These polarization values obtained in larger grain-sized ceramics can be attributed to the higher concentration of polar structures capable of rotation and switching, supported by the multidirectional polarization flexibility of the O phase compared to T phase.^37–40^ The field-induced positive strain (*S*_pos_) shows a modest increase from 0.22% to 0.34% between 0.9 and 11.1 μm grain-sized ceramics, whereas the negative strain (*S*_neg_) increases by an order of magnitude (*ca.* 0.04 to 0.43%). Such strain variation is not only attributed to non-180° domain wall motion^41,42^ but also field-induced phase transition between T and O phases near MPB.^25,43,44^ The effective high-field piezoelectric coefficient (*d*_33_*) obtained from the unipolar measurements was 1333, 1212, and 531 pm V^–1^ for samples with grain sizes of 0.9 μm, 5.4 μm, and 11.1 μm, respectively (Figure S5f). The increase in unipolar strain with grain size indicates a growing nonlinear electromechanical contribution, consistent with enhanced domain-wall activity and field-induced phase transitions.

**Figure 3:**
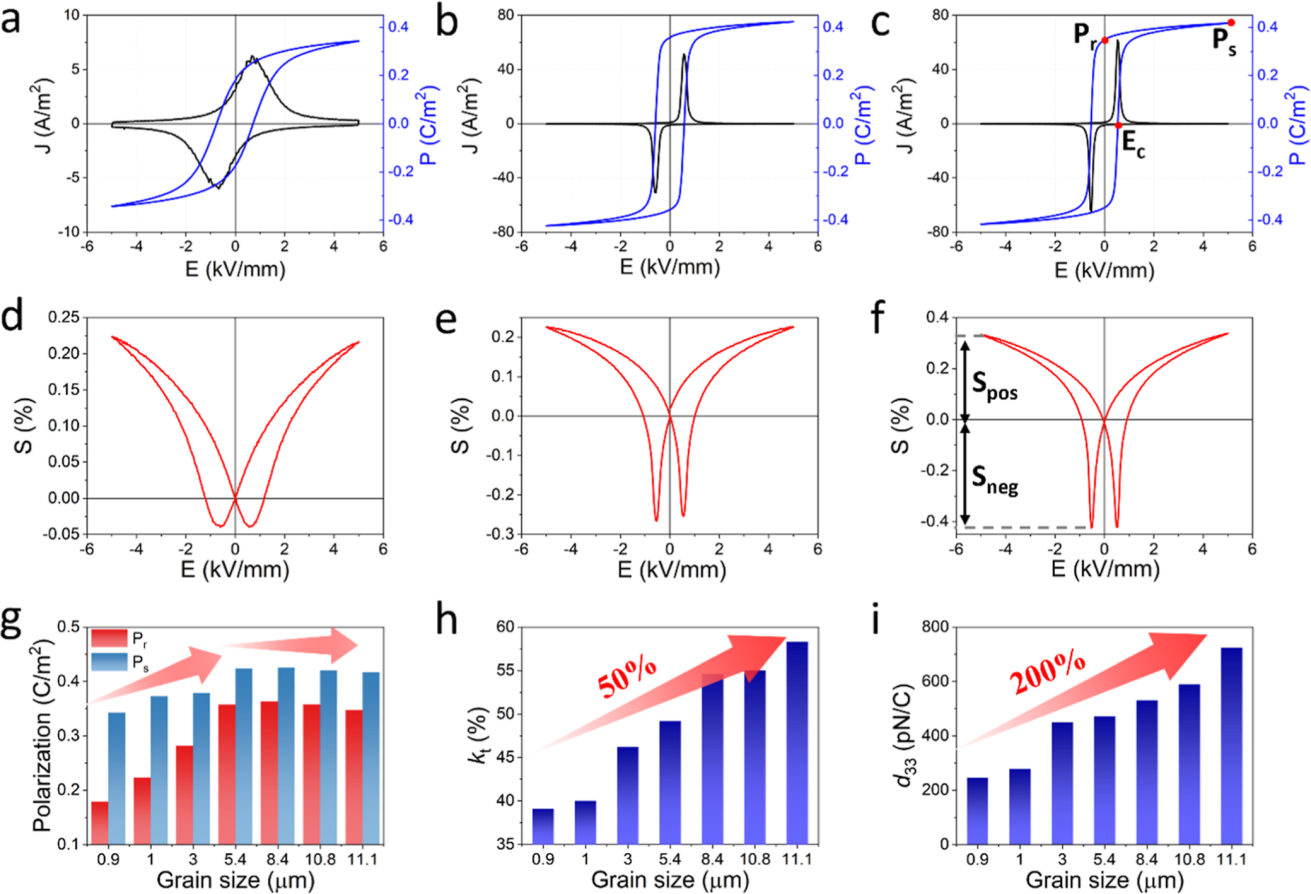
(a–f) *J*–*E*/*P*–*E*/*S*–*E* loops measured at 1 Hz for ErPMNT ceramics with grain sizes of (a,d) 0.9 μm, (b,e) 5.4 μm, and (c,f) 11.1 μm. Grain size dependence of (g) saturation polarization and remanent polarization, (h) piezoelectric coefficient *d*_33_, and (i) electromechanical coefficient *k*_t_ for ErPMNT ceramics.

The impedance and phase spectra, measured over the frequency range of 1 to 10 MHz for poled ceramics, are shown in Figure S6. The electromechanical coupling coefficient (*k*_t_) was calculated using the following equation.^45^

1 $$k_{t}^{2}=\frac{\pi f_{r}}{2f_{a}}\tan\left(\frac{\pi \left(f_{a}-f_{r}\right)}{2f_{a}}\right)$$

where resonance (*f*_r_) and antiresonance (*f*_a_) frequencies are the frequencies at impedance peak and valley points, respectively. Both *k*_t_ and piezoelectric coefficient *d*_33_ increase continuously as grain size grows even where P_s_ and P_r_ have plateaued. (Figure 3g–i). The *k*_t_ increases by 50% relative to the small grain-sized ceramic, reaching 58.3%, while *d*_33_ exhibits an impressive 200% increase to 723 pC N^–1^ in the 11.1 μm grain-sized ceramic. The sustained enhancement of electromechanical properties beyond the polarization plateau clearly demonstrates that the piezoelectric response is dominated by extrinsic contributions, where increased density and mobility of domain walls and the field induced phase transformation, rather than further increases in intrinsic lattice polarization.

The Raman spectra show gradual peak broadening and intensity variation with increasing grain size (Figure 4a), indicating enhanced local structural ordering and lattice symmetry modification. The Raman spectra of unpoled ErPMNT ceramics can be deconvoluted into nine distinct Raman modes and fitted using a Lorentzian peak function (Figures 4b and S7). These spectra are divided into three distinct regions based on their wavenumber ranges and corresponding structural vibrations: Region A (below 200 cm^–1^) is associated with the movement of A-site cations, Region B (200 to 400 cm^–1^) is linked to B–O bond vibrations, and Region C (above 400 cm^–1^) relates to the stretching and breathing vibration modes in the BO_6_ octahedra. The intensity of the B_2_ mode gradually decreases, while the B_1_ and B_3_ modes become more pronounced with increasing grain size. This phenomenon suggested to be associated with the ordering of the B-site, where a more ordered structure develops in larger-grained ceramics. The systematic shift of characteristic Raman modes (C_1_–C_5_) toward higher wavenumbers with increasing grain size (Figure 4c) indicates the increased stiffness of the local B–O bonding and reduced structural disorder.^46^ The normalized Raman spectra of the selected ceramics shown in Figure 4d–f provide insights into the structural changes between unpoled and poled states. For the 0.9 μm grain-sized ceramic, the Raman spectra exhibit almost identical patterns before and after poling, suggesting minimal structural changes during the poling process. This observation is consistent with the low remanent polarization observed in the *P*–*E* loops. In contrast, notable changes in the Raman spectra were observed for larger grain-sized ceramics (5.4 and 11.1 μm) after poling. Specifically, the peak intensity in the 300–600 cm^–1^ range was significantly increased after poling, particularly in the B_3_, C_1_, and C_2_ modes, with the effect being more pronounced in larger grain-sized ceramics. The observed narrowing of the 700–800 cm^–1^ peak suggests increased structural ordering in larger grain-sized ceramics, which correlates with the reduced tetragonal phase fraction and enhanced domain alignment.^47,48^ These changes are associated with the field-induced transition from local tetragonal to orthorhombic phases as well as the alignment of the domain structure. Such spectral variation between unpoled and poled states is consistent with the large remanent polarization seen in the *P*–*E* loops. To further examine the influence of poling process, XRD patterns of ceramics in the unpoled and poled states were compared (Figure 4g–i). While no new diffraction peaks appear after poling, subtle intensity changes can be observed in the pseudocubic {220}_pc_ diffraction. For the 0.9 μm grain-sized ceramic, the {220}_pc_ pattern remained nearly identical before and after poling. In contrast, the larger grain-sized ceramics exhibited noticeable peak shifts and changes in peak profiles. These differences indicate that poling alters the relative concentration of tetragonal and orthorhombic phases, consistent with the expected field-induced phase transition at the MPB. Due to the strong texture effects introduced by the SPS process and the significant overlap between T and O reflections, detailed Rietveld refinement of ceramics was not feasible. Nevertheless, the qualitative comparison clearly shows a structural change due to poling, consistent with the Raman spectra.

**Figure 4:**
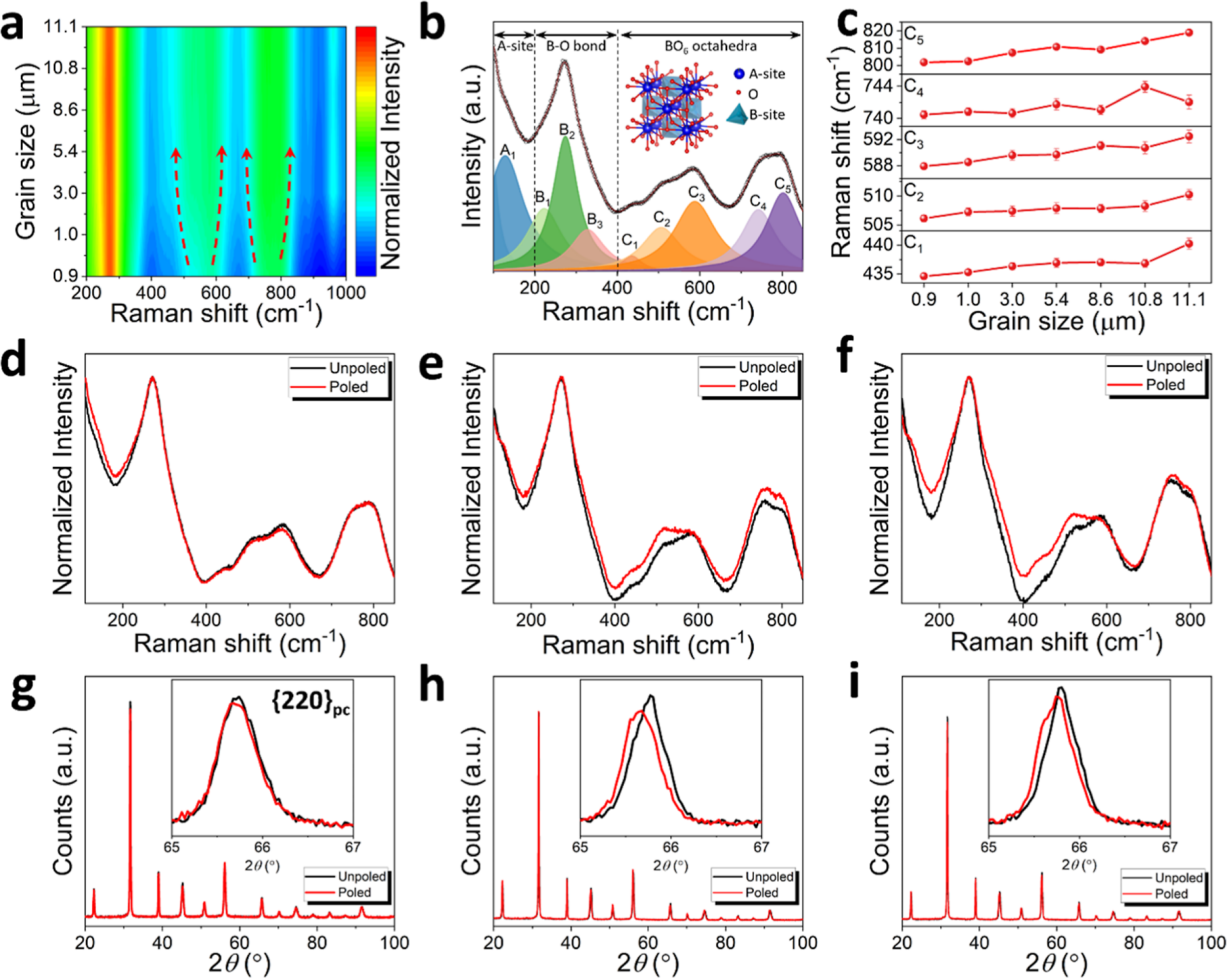
(a) Contour plot of Raman spectra for ErPMNT ceramics with different grain sizes. (b) Fitted Raman spectrum of ErPMNT with a grain size of 0.9 μm, showing multipeak deconvolution and corresponding vibrational mode assignments associated with A-site, B-site, and BO_6_ octahedral contributions. (c) Evolution of characteristic Raman peak positions (C_1_–C_5_) for studied ceramics. Raman spectra of unpoled and poled ErPMNT ceramics with grain sizes of (d) 0.9 μm, (e) 5.4 μm, and (f) 11.1 μm. XRD patterns of unpoled and poled ErPMNT ceramics with grain sizes of (g) 0.9 μm, (h) 5.4 μm, and (i) 11.1 μm.

Piezoresponse force microscopy (PFM) was employed to investigate the domain structures of ErPMNT ceramics with grain sizes of 0.9 μm, 5.4 μm, and 11.1 μm (Figures 5 and S8). All ceramics exhibited smooth surfaces, with root-mean-square roughness (*R*_q_) values of 3.81, 1.79, and 1.02 nm, respectively. In the 0.9 μm grain-sized ceramic, the amplitude image shows weak piezoelectric signal, while the phase image reveals irregular and diffused polarization patterns. This suggests that the smaller grain size limits the domain growth and ordering. In contrast, the 5.4 and 11.1 μm grain-sized ceramics exhibit clear ferroelectric domain structures with clear amplitude and phase contrast, indicating well developed domain walls and polar regions. The 5.4 μm ceramic exhibited a higher domain wall density, as seen in the amplitude image, with numerous fine contrast lines forming a densely packed network of ferroelectric domains. The 11.1 μm grain-sized ceramic exhibits thicker and distinct domain walls, forming a web-like domain network with broader spacing between walls. Under a ±10 V DC bias applied via conductive tip, a square region with well-defined phase reversal was formed, as highlighted by the dashed yellow box. In the 0.9 μm grain-sized ceramic, amplitude and phase response were weak and scattered, indicating diffused domains and low polar ordering. In contrast, the 5.4 and 11.1 μm grain-sized ceramics show a sharp 180° phase shift and corresponding amplitude drop, confirming reversible polarization switching. This amplitude depression at the domain boundary was attributed to the reduced piezoresponse due to domain wall pinning and local mechanical clamping. With increasing grain sizes, domain contrast becomes more distinct and the switched region more stable, indicating enhanced ferroelectric ordering and improved domain mobility. The domain wall density was higher in 5.4 μm grain-sized ceramics compared to the 11.1 μm grain-sized ceramics. Domain wall widths extracted from line profiles were approximately 150 nm (5.4 μm grain) and 400 nm (11.1 μm grain). These results suggest that while domain wall density decreases with increasing grain size, domain wall thickness increases, leading to stronger polarization coupling and broader strain accommodation in larger grains.

**Figure 5:**
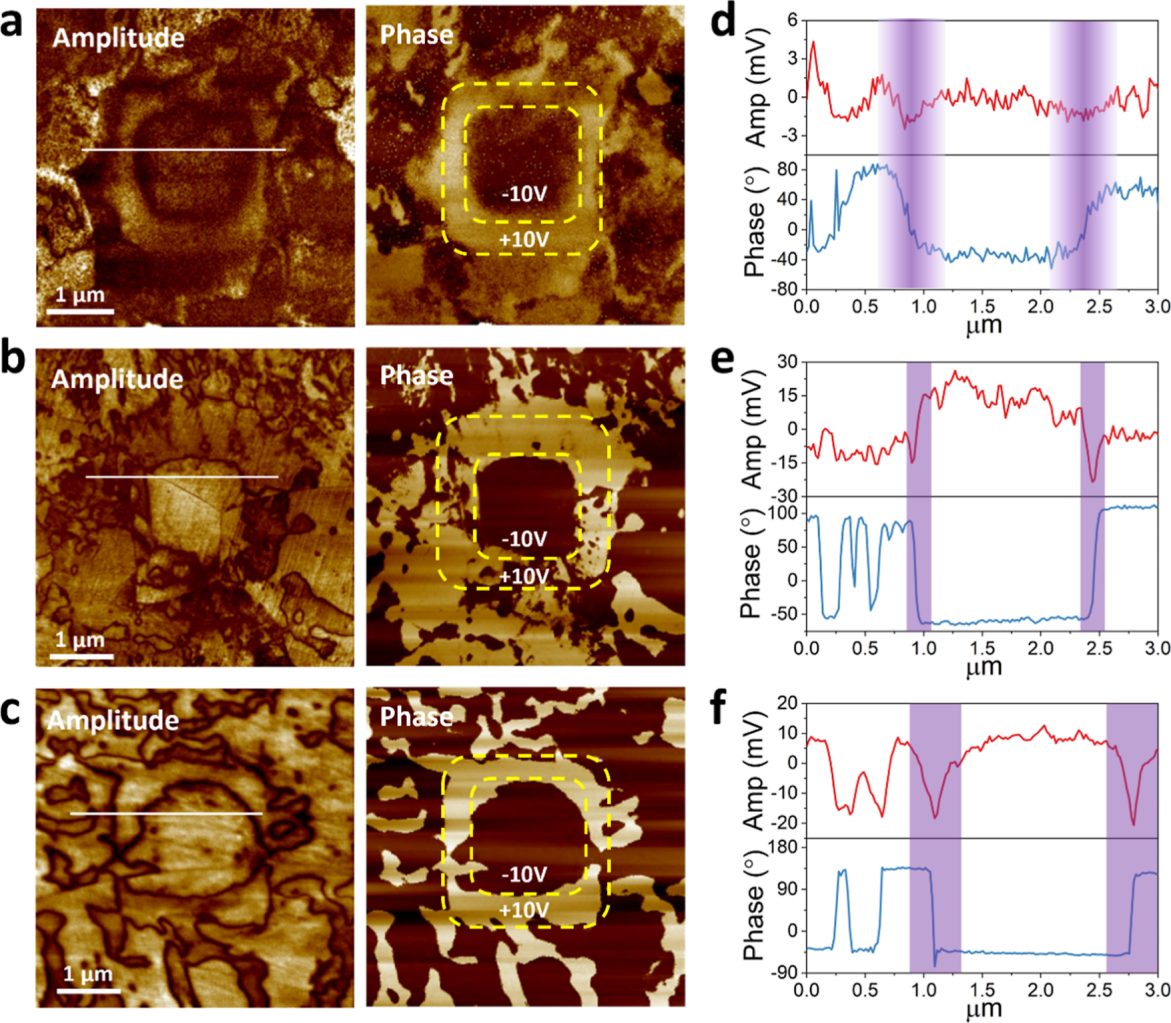
PFM amplitude and phase images of ErPMNT ceramics with grain sizes of (a) 0.9 μm, (b) 5.4 μm, and (c) 11.1 μm after local poling under a ±10 V DC bias (yellow dashed boxes). (d–f) Corresponding line profiles taken along the white dashed lines in (a–c), with domain walls highlighted by the purple-shaded regions.

The FE domain structure, which is closely linked to the crystal structure, has a significant influence on the dielectric and piezoelectric properties of PMN–PT relaxor ferroelectrics.^23^ Transmission electron microscopy (TEM) was used to explore the local domain configurations of ErPMNT ceramics with grain sizes of 0.9, 5.4, and 11.1 μm (Figures 6 and S9). The 0.9 μm grain-sized ceramic exhibits a distinctive “core–shell” structure. The shell regions contain lamellar domains with widths ranging from *ca.* 50 to 100 nm, while the core regions show more diffuse features, suggesting suppressed long-range domain development. Such core–shell domain morphologies have also been observed in lead titanate or bismuth sodium titanate-based relaxor ferroelectrics,^49,50^ and are generally associated with local stress or defect–related nanoscale heterogeneity. Such microstructural complexity in the system restricts domain wall mobility and contributes to the formation of rigid “constrained domain walls”. Such constrained walls are more susceptible to pinning, thereby limiting domain switching and contributing to higher coercive fields and lower remanent polarization values, as well as the smaller piezoelectricity and weaker signal in the corresponding phase images. In 5.4 μm grain-sized ceramic, more complex and dense domain structures were observed, with increased density of well-defined, nanoscale domains within the grain. These domains exhibited different orientations, forming interconnected, stripe-like configurations that extended across multiple nanoregions. The higher domain wall density with finer structural features indicates the emergence of more flexible domain walls that are less affected by grain boundary pinning. This enhanced domain wall mobility is beneficial to reversible domain switching and leads to improved electromechanical response, consistent with the sharp polarization reversal in PFM. In the coarse-grained ceramic (11.1 μm), the domain structure transforms into a well-organized, hierarchical architecture with a coexistence of striation-like nanodomains and wedge-shaped microscale domains, particularly concentrated near grain boundaries. This multiscale domain configuration indicates a relaxation of internal stress, allowing the development of larger and more energetically stable polarization states. Notably, the domains exhibit flux-closure loops at junctions and boundaries, effectively minimizing depolarization fields and stabilizing local polarization states.

**Figure 6:**
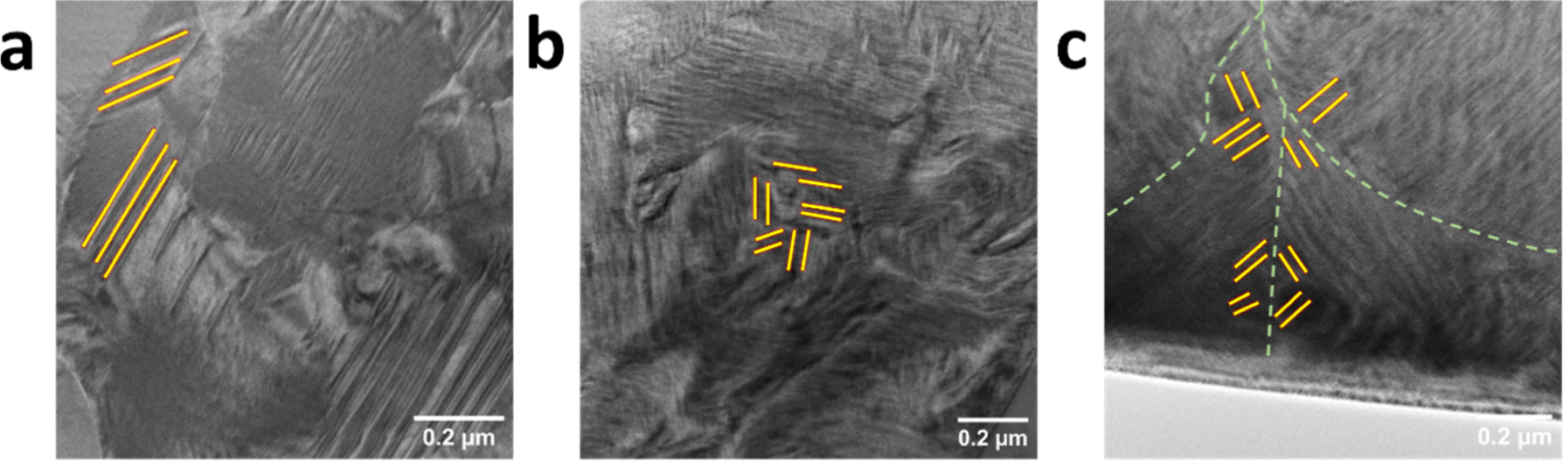
(a) TEM images of ErPMNT ceramics with (a) 0.9 μm, (b) 5.4 μm, and (c) 11.1 μm grain sizes.

It is suggested that domain evolution progresses from a constrained, short-range ordered state in small grains to dense, fine, and mobile domain walls in medium-sized grains and finally to hierarchical domain networks with thicker walls and local relaxation in large grains. These trends are consistent with reports that strain fields at grain boundaries and domain walls modify wall mobility and stability in ferroelectrics.^51–53^ Grain-size effect on phase coexistence reveals that smaller grains can preserve a fraction of T phase in an O phase matrix upon cooling due to the internal stress induced by grain boundaries.^54,55^ Within these small domains, the localized dipole arrangements may form around distorted O-phase polar regions in order to mitigate depolarization fields.^56^ In contrast, larger grain-sized ceramics exhibit larger, well-ordered domains with complex configurations and flexible, “mobile domain walls”. Within the grain, tetragonal FE dipoles primarily localize at domain corners and tend to form flux–closure loops at domain edges to minimize depolarization field effects.^57–59^ Low random pinning within grains lowers the coercive field and improves domain wall mobility. Meanwhile, localized defect pinning enhances the stability of highly ordered domain post-poling, resulting in high saturation and remanent polarization values. These values increase with grain size up to approximately 5.4 μm, beyond which they plateau, which can be attributed to several factors related to saturation of domain wall mobility, internal stress balance, and intrinsic limitation of polarization alignment. In addition, ferroelectric domain walls in large-grain-sized ceramics are more flexible and contain more ordered domain structure, which leads to continuously increasing field-induced strain, *k*_t_ and *d*_33_ values.

## 3 Conclusion

This systematic study investigates dense ErPMNT ceramics with grain sizes ranging from 0.9 to 11.1 μm, achieved through controlled SPS conditions. XRD analysis confirmed the coexistence of tetragonal and orthorhombic phases, with smaller grains showing a higher tetragonal fraction due to grain boundary-induced stress. As grain size increases, progressive stress relaxation increases orthorhombic phase fraction and enhanced domain wall mobility. Correspondingly, the dielectric permittivity of small grain-sized ceramics exhibits a diffuse dielectric behavior, whereas larger grains show sharp dielectric peaks with increased permittivity and reduced *T*_m_, indicating improved ferroelectric ordering. Ferroelectric measurements revealed that both saturation and remanent polarization increased with grain size up to 5.4 μm and then plateaued. In contrast, electromechanical properties continued to improve throughout the grain size range, with the piezoelectric coefficient (*d*_33_) increased by ∼200% to 723 pC N^–1^, and the coupling factor (*k*_t_) rose by ∼50 to 58.3% for the largest grain-sized ceramics. PFM and TEM analysis reveal that in small grain-sized ceramics, disordered core–shell structures predominate, exhibiting limited mobility due to internal stress and defect pinning. At intermediate grain size, dense nanodomain networks emerge with narrow domain walls approximately 150 nm wide, exhibiting enhanced wall sensitivity and sharp polarization switching. In large grain-sized ceramics, domain structures transform into hierarchical, web-like patterns with broader spacing and thicker walls around 400 nm. These domains exhibit increased mobility and the formation of flux–closure loops at domain junctions and boundaries, which serve to minimize depolarization fields and stabilize polarization configurations. The results demonstrate that grain-size engineering effectively tunes domain wall dynamics and phase stability, offering a practical pathway to achieve superior piezoelectric and ferroelectric performance in relaxor-based ceramics for advanced energy, sensing, and actuation applications.

## Supplementary Material



## Notes

SEM images, refined XRD profiles, *P*–*E*/*J*–*E* loops, impedance spectra, fitted Raman spectra, PFM images, TEM images, and details of XRD refined parameters (PDF)
