# Supplementary material for: Grain Size-Dependent Defect and Domain Evolution in Lead Titanate-Based Relaxor Ferroelectrics
Source: ACS Appl Mater Interfaces. 2026 Apr 22;18(17):24889–98. doi: 10.1021/acsami.6c00336 (PMC13154116; doi:10.1021/acsami.6c00336)
Supplement: Supplementary file 1 [file am6c00336_si_001.pdf]

`

Supporting Information

Grain size-dependent defect and domain evolution in lead titanate-based relaxor ferroelectrics

Hangfeng Zhang, [1] *Yichen Wang,*[2] *Zilong Li,*[1] *Soyoung Oh,*[3] *Junjie Liu,*[4] *Haixue Yan,*[*1] *Yang Hao,*[*5] *Lei Su*[*1]

[1] School of Engineering and Material Science, Queen Mary University of London, Mile End Road, London E1 4NS, UK.

[2] State Key Laboratory of Powder Metallurgy, Central South University, Changsha 410083, China

[3] Department of Physics, University of Oxford, Oxford, OX1 3PU, UK

[4] School of Physical and Chemical Science, Queen Mary University of London, Mile End Road, London E1 4NS, UK.

[5] School of Electronic Engineering and Computer Science, Queen Mary University of London, Mile End Road, London E1 4NS, UK.

E-mail addresses: h.x.yan@qmul.ac.uk (H. Yan), y.hao@qmul.ac.uk (Y. Hao), l.su@qmul.ac.uk (L. Su).

`

Experimental Section

Material Synthesis. ErPMNT powder was synthesized using a two-step solid state method.[1] In the first step, stoichiometric amounts of magnesium oxide (MgO, 99%, Sigma Aldrich) and niobium oxide (Nb_2O_5, 99.9%, Sigma Aldrich) were mixed and ball milled in ethanol at 180 rpm for 20 h using a planetary ball mill (Fritsch Pulverisette 5/4 classic line, Germany) with zirconia balls. The resultant mixture was then dried and subsequently calcined in an alumina crucible at 1200 °C for 2 h, forming $MgNb_2O_6$. In the second step, stoichiometric amounts of erbium oxide (Er_2O_3, 99.9%, Sigma Aldrich), lead oxide (Pb_3O_4, 99%, Sigma Aldrich), titanium oxide (TiO_2, 99.8%, Sigma Aldrich) and $MgNb_2O_6$ were ball milled in ethanol at 180 rpm for 20 h, followed by drying and sieving through a 250 μm mesh. The sieved mixture underwent calcination at 840 °C for 4 h. After cooling, the calcined powder was ball milled again, dried and sieved. Subsequently, ceramics with varying grain sizes were prepared using SPS (SPS, HPD 25/1, FCT, Rauenstein, Germany) at temperatures ranging from 900 to 1200 °C for 2 to 30 min, under an applied uniaxial pressure of 60 MPa. The SPS ceramics were cut into thin slices and annealed at 800 °C for 20 h to remove residual carbon and release internal stress. The density of final pellets was measured by the Archimedes method.

Material Characterization. For X-ray powder diffraction (XRD) analysis, the thin ceramics were gentle grounded into fine powder. XRD measurements were conducted using a PANalytical CUBIX3 diffractometer, which is equipped with an X'Celerator detector and utilizes Ni filtered Cu-Kα radiation (λ = 1.5418 Å). XRD data were collected over the 2θ range of 5-120°, with a step width of 0.0334° and an effective count time of 200s per step. The measured data were modeled by Rietveld analysis using the GSAS suite of programs.[2] Structure refinement was carried out with initial tetragonal and orthorhombic models in space groups *P*4*mm* and *Amm*2, respectively.[3] Scanning electronic microscopy (SEM, FEI Inspect F) images were taken on the fresh fracture surface of the ceramic sample with an incident beam of 30 kV. Measurements were first conducted on a poled sample surface, followed by annealing at 350°C for 1 hour to induce depoling. After cooling, Raman spectra were remeasured on the same sample surface to compare the poled and unpoled states. Piezoresponse force microscopy (PFM) was conducted using an atomic force microscopy (AFM) system (Bruker Dimension Icon, US) equipped with an SCM-PIT-V2 conductive probe (Bruker, US). Ceramic samples were sequentially polished using silicon carbide grinding paper with 1200, 2400, and 4000 grit, followed by further polishing with diamond suspension of 6 μm, 3 μm, 1 μm, and 1/4 μm grain size to achieve a smooth surface. The polished samples were then annealed at 400°C for 3 hours to relieve residual stress and stabilize the domain structure. A ceramic sample was ground

down to approximately 100 μm in thickness, followed by dimple grinding on the disc. Final thinning to electron transparency was achieved using Ar-ion milling at 2 kV with a Gatan PIPS system. Domain structure was characterized in Transmission electron microscopy (TEM, JEM-F200, Japan).

Electrical measurements. Silver pastes (Gwent Electronic Materials Ltd. Pontypool, U.K.) were applied to ErPMNT ceramic surfaces and then heated at 500°C for 20 mins to form conductive layers. Dielectric properties were measured at room temperature using a precision impedance analyser (Agilent 4294A). Temperature dependence of dielectric properties was measured using an LCR meter (Agilent 4284A) and a temperature control chamber. Ferroelectric properties, including current density-electric field (*J-E*), polarization-electric field (*P-E*) and strain-electric field (*S-E*) were measured on samples with a thickness of 0.40-0.45 mm using a ferroelectric hysteresis tester (NPL, UK), applying a triangle voltage waveform with an AC electric field of 5 kV mm^{-1}. [4] Electrical poling of the sample involved applying a 4kV mm^{-1} DC electric field for 5 min in silicon oil. The piezoelectric coefficient (d_{33}) was measured with a quasi-static ZJ-3B d_{33} meter (Institute of Acoustics Academia Sinica, China).

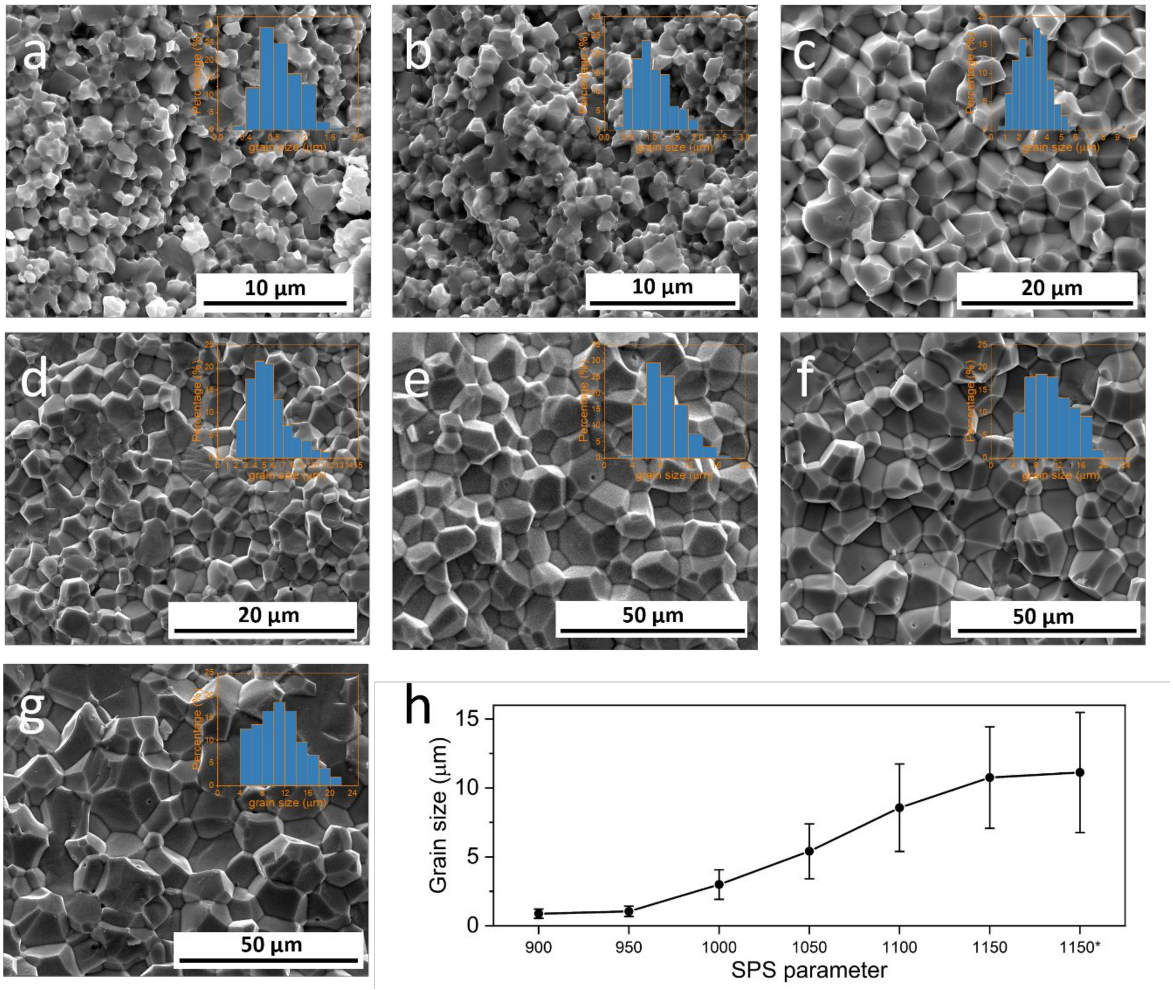

Figure S1. SEM images of fresh cross-section morphology with the corresponding grain size distribution histograms (inset) for studied ErPMNT ceramics , (a) 900°C/2m, (b) 950°C/2m, (c) 1000°C/2m, (d) 1050°C/2m, (e) 1100°C/2m, (f) 1150/2m and (g) 1150°C/5m. (h) Average grain size with error bar for samples with different SPS conditions.

`

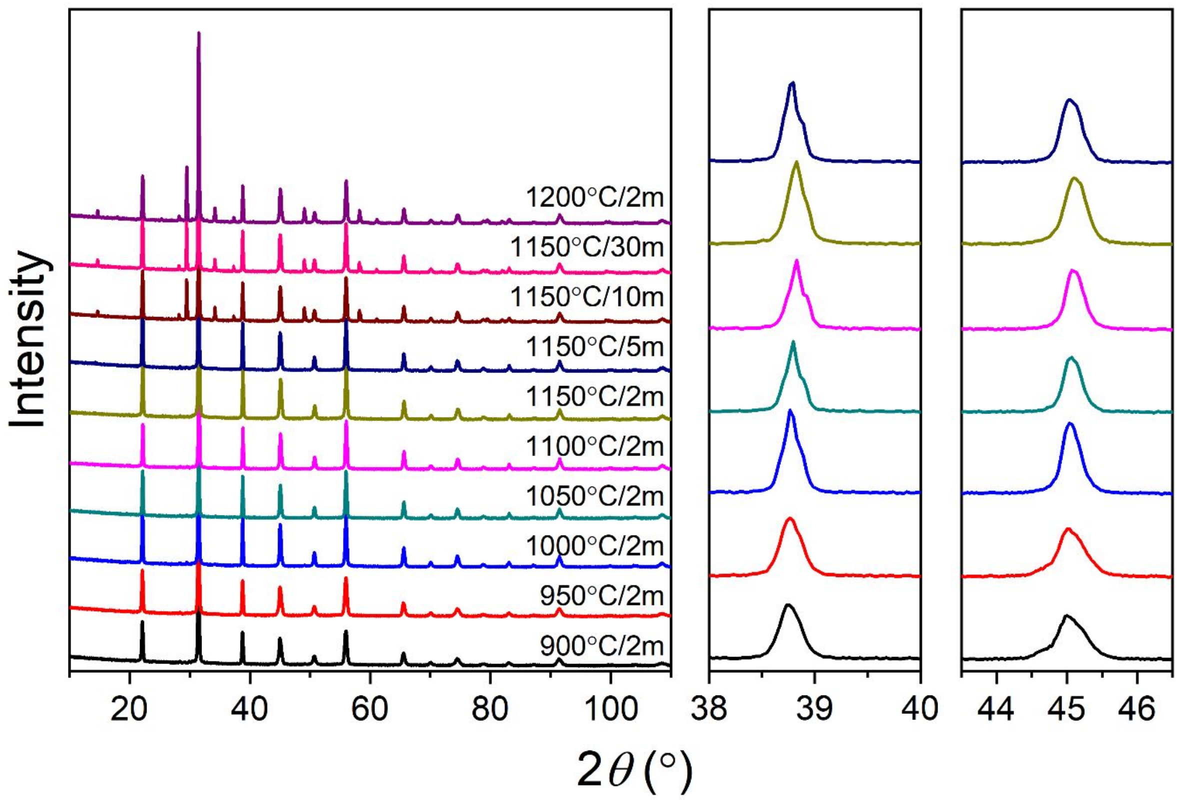

Figure S2. XRD patterns of ErPMNT ceramics sintered at different SPS conditions.

`

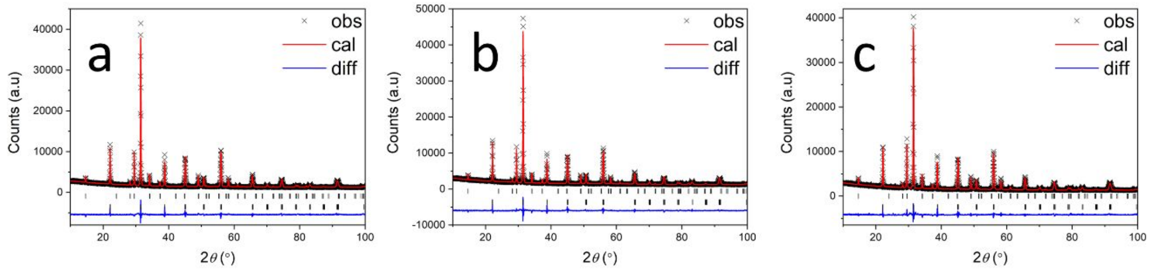

Figure S3. Fitted XRD profiles for ErPMNT ceramics sintered at (a) 1150 °C for 10min, (b) 1150 °C for 30min and (c) 1200 °C for 10 min.

`

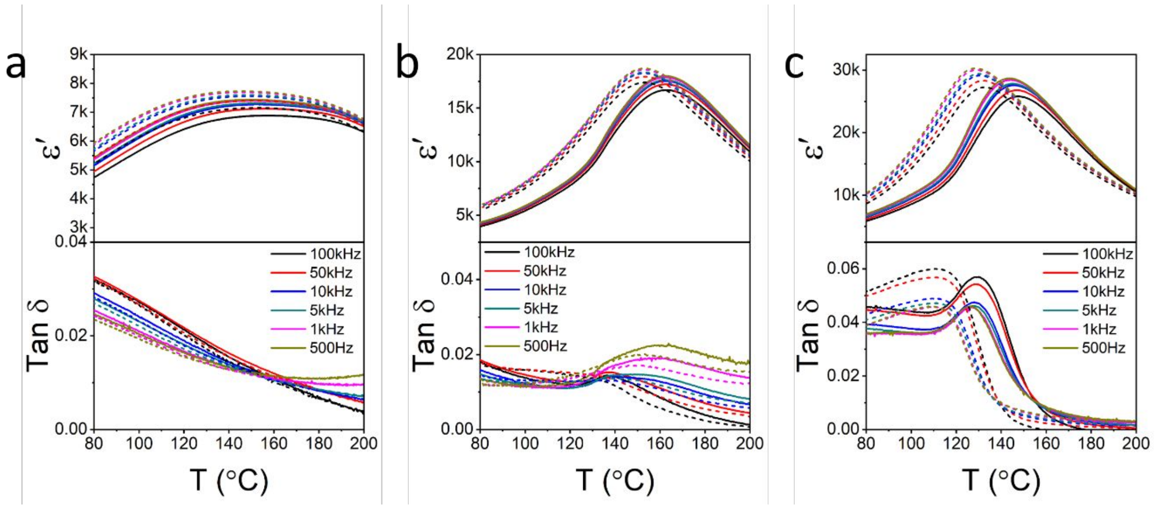

Figure S4. Temperature dependence of dielectric permittivity and loss of ErPMNT ceramics with grain sizes of (a) 0.9 μm, (b) 5.4 μm and (c) 11.1 μm measured at selected frequencies. solid line and dashed curves correspond to heating and cooling cycles, respectively.

`

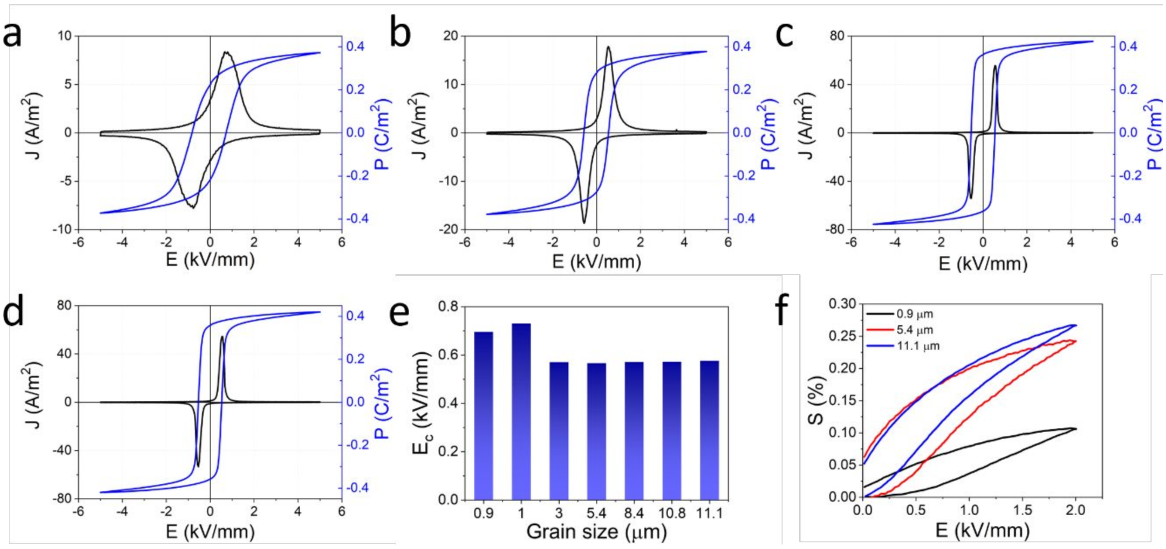

Figure S5. *J-E*/*P-E* loops measured at 1Hz for (a) 1.0 μm, (b) 3.0 μm, (c) 8.6 μm and (d) 10.8 μm grain sized ErPMNT ceramics. (e) Grain size dependence of coercive field for studied ceramics. (f) Unipolar *S-E* loops measured at 1Hz for selected ceramics.

`

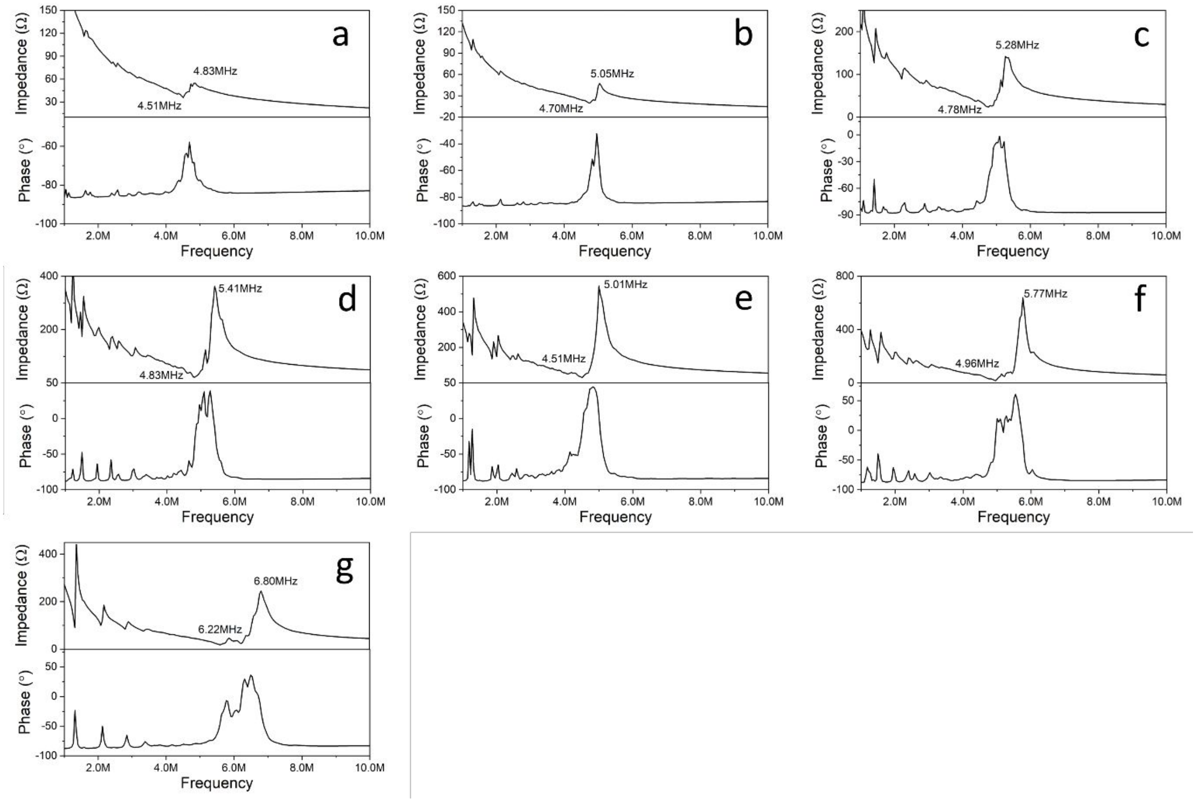

Figure S6. Frequency dependence of impedance and phase for ErPMNT ceramic samples with different grain sizes, (a) 0.9µm, (b) 1.0 µm, (c) 3.0 µm, (d) 5.4 µm, (e) 8.6 µm, (f) 10.8 µm and (g) 11.1µm.

`

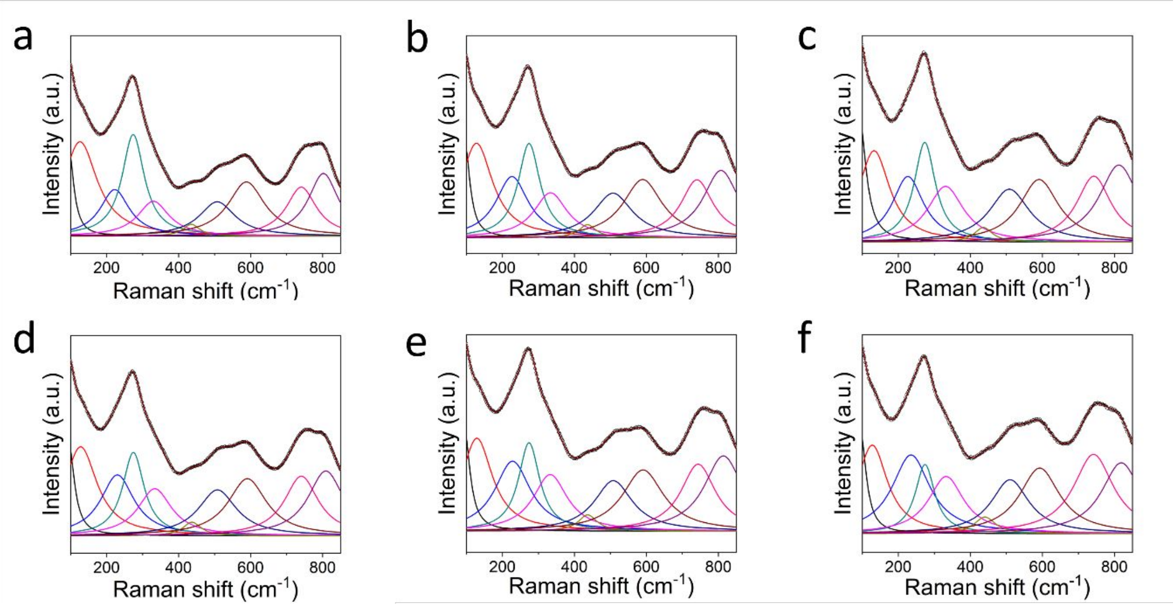

Figure S7. Fitted Raman spectra for ErPMNT ceramic samples with different grain sizes, (a) 1.0 µm, (b) 3.0 µm, (c) 5.4 µm, (d) 8.6 µm, (e) 10.8 µm and (f) 11.1µm.

`

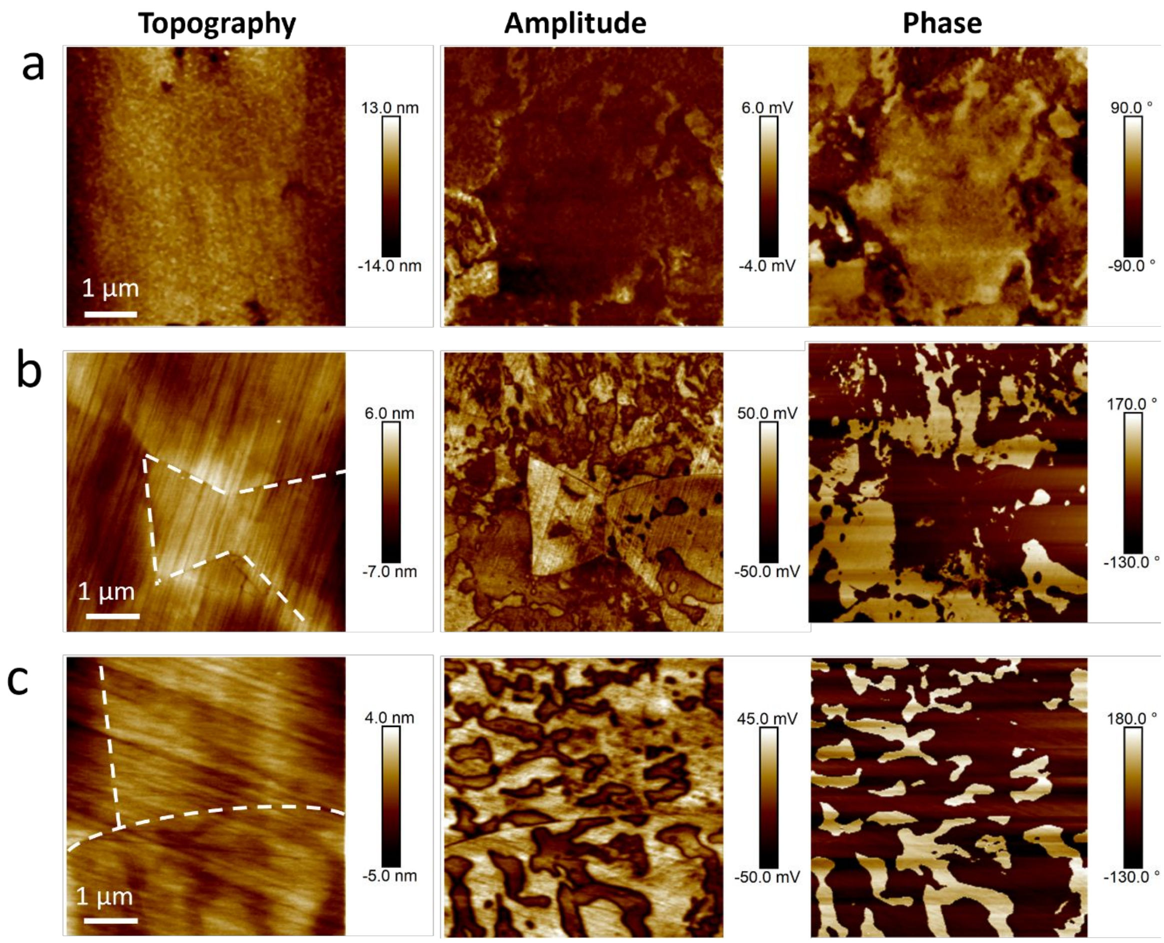

Figure S8. PFM topography, amplitude and phase images of unpoled ErPMNT ceramic samples with (a) 0.9 µm, (b) 5.4 µm and (c) 11.1 grain sizes.

`

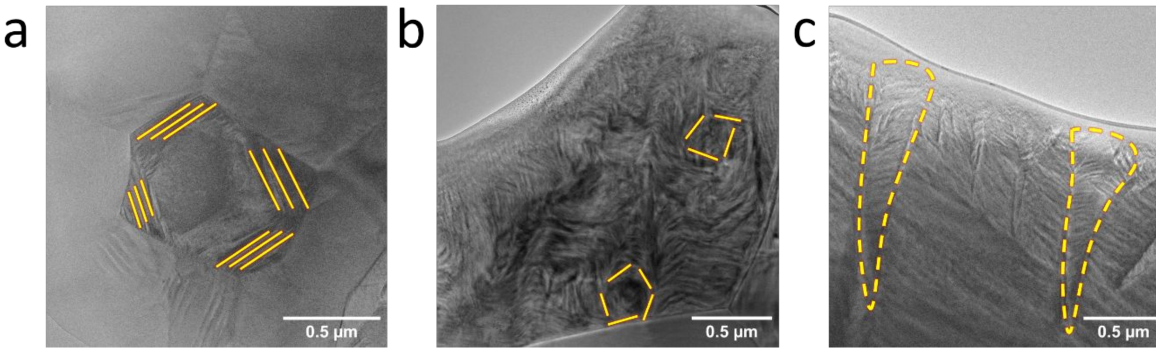

Figure S9. (a) TEM images of ErPMNT ceramic samples with (a) 0.9 µm, (b) 5.4 µm and (c) 11.1 µm grain sizes.

`

Table S1. Crystal and refinement parameters for ErPMNT ceramic samples with different grain sizes at room temperature. Estimated standard deviations are given in parentheses.

**Grain size (μm)**	**0.9**	**1.0**	**3.0**	**5.4**	**8.6**	**10.8**	**11.1**
**Space group**	*P*4*mm*	*P*4*mm*	*P*4*mm*	*P*4*mm*	*P*4*mm*	*P*4*mm*	*P*4*mm*
**Phase fraction**	40.55%	37.84%	15.59%	15.35%	13.36%	13.42%	11.97%
**Unit cell dimensions (Å)**	*a* = 4.0077(2)<br>*c* = 4.0620(3)	*a* = 4.0080(2)<br>*c* = 4.0607(4)	*a* = 4.0257(4)<br>*c* = 4.0449(7)	*a* = 4.0267(5)<br>*c* = 4.0457(8)	*a* = 4.0304(6)<br>*c* = 4.0432(9)	*a* = 4.0235(3)<br>*c* = 4.0487(6)	*a* = 4.0242(4)<br>*c* = 4.0413(7)
**Volume (Å^3)**	65.246(7)	65.232(8)	65.55(1)	65.60(2)	65.68(2)	65.54(1)	65.44(1)
**Z**	1	1	1	1	1	1	1
**D_{calc} (g cm^{-3})**	8.017	8.019	7.979	7.974	7.964	7.980	7.992
**Space group**	*Amm*2	*Amm*2	*Amm*2	*Amm*2	*Amm*2	*Amm*2	*Amm*2
**Phase fraction**	59.45%	62.16%	84.41%	84.65%	86.64%	86.58%	88.03%
**Unit cell dimensions (Å)**	*a* = 4.0183(2)<br>*b* = 5.70(1)<br>*c* = 5.70(1)	*a* = 4.0184(2)<br>*b* = 5.698(1)<br>*c* = 5.711(1)	*a* = 4.0176(1)<br>*b* = 5.701(1)<br>*c* = 5.701(1)	*a* = 4.0181(1)<br>*b* = 5.7002(7)<br>*c* = 5.7029(8)	*a* = 4.0180(1)<br>*b* = 5.6991(4)<br>*c* = 5.7041(6)	*a* = 4.0145(1)<br>*b* = 5.699(1)<br>*c* = 5.699(1)	*a* = 4.0143(1)<br>*b* = 5.7023(4)<br>*c* = 5.6968(3)
**Volume (Å^3)**	130.82(1)	130.80(1)	130.596(1)	130.622(8)	130.62(1)	130.41(1)	130.40(1)
**Z**	1	1	2	2	2	2	2
**D_{calc} (g cm^{-3})**	7.997	7.998	8.011	8.009	8.009	8.022	8.022
**R-factors$^{\alpha}$**	R_{wp} = 0.0341<br>R_p = 0.0263<br>R_{ex} = 0.0232<br>R_F^2 = 0.0523<br>χ^2 = 2.114	R_{wp} = 0.0349<br>R_p = 0.0269<br>R_{ex} = 0.0233<br>R_F^2 = 0.0469<br>χ^2 = 2.258	R_{wp} = 0.0373<br>R_p = 0.0286<br>R_{ex} = 0.0228<br>R_F^2 = 0.0392<br>χ^2 = 2.681	R_{wp} = 0.0369<br>R_p = 0.0279<br>R_{ex} = 0.0243<br>R_F^2 = 0.0463<br>χ^2 = 2.326	R_{wp} = 0.0373<br>R_p = 0.0284<br>R_{ex} = 0.0244<br>R_F^2 = 0.0379<br>χ^2 = 2.356	R_{wp} = 0.0364<br>R_p = 0.0275<br>R_{ex} = 0.0236<br>R_F^2 = 0.0347<br>χ^2 = 2.621	R_{wp} = 0.0366<br>R_p = 0.0273<br>R_{ex} = 0.0230<br>R_F^2 = 0.0513<br>χ^2 = 2.542
**No. of variables**	30	30	28	28	28	28	28
**No. of profile points**	3649	3649	3649	3649	3649	3649	3649

$^{\alpha}$For definition of R-factors see reference [2].

`

Reference,

(1) Swartz, S. L.; Shrout, T. R. Fabrication of Perovskite Lead Magnesium Niobate. *Mater. Res. Bull.* **1982**, *17* (10), 1245–1250.

(2) Larson, A. C.; Dreele, R. B. *Los Alamos National Laboratory Report*; 1987.

(3) Li, F.; Lin, D.; Chen, Z.; Cheng, Z.; Wang, J.; Li, C.; Xu, Z.; Huang, Q.; Liao, X.; Chen, L. Q.; Shrout, T. R.; Zhang, S. Ultrahigh Piezoelectricity in Ferroelectric Ceramics by Design. *Nat. Mater.* **2018**, *17* (4), 349–354..

(4) Viola, G.; Saunders, T.; Wei, X.; Chong, K. B.; Luo, H.; Reece, M. J.; Yan, H. Contribution of Piezoelectric Effect, Electrostriction and Ferroelectric/Ferroelastic Switching to Strain-Electric Field Response of Dielectrics. *J. Adv. Dielectr.* **2013**, *03* (01), 1350007.
